# Particle Imaging Velocimetry with Color-Encoded Illumination: A Review

**DOI:** 10.3390/s25164981

**Published:** 2025-08-12

**Authors:** Yizhu Wang, Xiaoming He, Yuan Tian, Chang Liu, Depeng Wang

**Affiliations:** 1 College of Energy and Power Engineering, Nanjing University of Aeronautics and Astronautics, Nanjing 210016, China; wangyizhu@nuaa.edu.cn (Y.W.); vehicle@nuaa.edu.cn (X.H.); 2 Department of Biomedical Engineering, Duke University, Durham, NC 27708, USA

**Keywords:** particle image velocimetry, color-encoded illumination, flow measurement

## Abstract

High-resolution and three-dimensional measurements at large scales represent a crucial frontier in flow diagnostics. Color-encoded illumination particle imaging velocimetry has emerged as a promising non-contact volumetric measurement technique in recent years. By employing chromatic gradient illumination to excite tracer particles, this method encodes depth information into color signatures, which are then correlated with two-dimensional positional data in images to reconstruct three-dimensional flow fields using a single camera. This review first introduces the fundamental principles of particle image velocimetry/particle tracking velocimetry and chromatic-depth encoding. Subsequently, we categorize color-depth-encoded particle velocimetry methods based on different illumination strategies, including LED-based, projector-based, and laser-based systems, discussing their respective configurations and representative applications. Finally, we summarize the current research progress in color-encoded particle image velocimetry techniques, provide a comparative analysis of their advantages and limitations, and discuss existing challenges along with future development prospects.

## 1. Introduction

Fluid motion is one of the most ubiquitous phenomena in nature, and its research prerequisite lies in obtaining accurate quantitative flow field velocity information [1]. Based on the motion of small tracer particles seeded in a fluid, particle image velocimetry (PIV) and particle tracking velocimetry (PTV) provide powerful experimental means to quantify flow velocity. It has become an essential tool in fluid mechanics research and has provided detailed and accurate velocity information crucial for understanding flow phenomena, validating numerical simulations, and optimizing engineering designs. The basic principle of PIV assumes that the small size and low inertia tracer particles follow the fluid motion closely. Flow velocity is calculated by recording particles’ positions at different time points [2]. The flow imaging applications extend significantly through the disciplines of science and engineering. Related studies include imaging phenomena such as flames [3,4], smoke [5,6], and fluid mixtures [7]. Interdisciplinary applications are also becoming increasingly extensive, and it is increasingly used in precise flow measurement and research in fields such as microfluidics [8], biomechanics [9,10], and nanomedicine [11]. Despite achieving volumetric reconstructions, existing techniques provide only timestamp-specific density scalar fields. Comprehensive analysis of 3D flows and advancement beyond basic visualization, however, necessitates the additional acquisition of 3D velocity fields. Consequently, the ultimate task is to obtain three-dimensional three-component (3D-3C) velocity vectors. Over many years, related techniques have been continuously evolving. Improvements in laser technology, camera sensitivity, and data processing algorithms have led to higher resolution measurements, faster acquisition rates, and more accurate results.

High-resolution and large-scale 3D-3C PIV is now a crucial direction of exploration in flow imaging. The recent advances in color-encoded illumination have greatly simplified PIV systems. By using illuminated particles with a color gradient, color information is encoded to correspond with the depth particles position in three-dimensional flow. When combined with two-dimensional positional data from images, this approach enables the reconstruction of 3D velocity fields with a monocular setup. In this review, we present the fundamental principles of color-encoded illumination-based PIV and highlight its unique capabilities in single-camera 3D imaging and high-precision flow diagnostics. While color-encoded PIV demonstrates promising potential, its broader application requires systematic development and standardization to fully unlock its ability in future fluid dynamics research. Current research mostly focuses on a specific lighting technology, but lacks systematic sorting of fundamental issues, such as the applicable boundaries of different lighting strategies. This review aims to construct a coherent knowledge framework. We illustrate the designs and applications of color-encoded PIV with different illumination strategies, including LED-based, projector-based, and laser-based illumination systems. Concludingly, we discuss the prevailing challenges and prospects for color-encoded PIV, providing further development pathways for the systems.

## 2. Fundamentals

### 2.1. Particle Image Velocimetry (PIV)

In PIV, a laser sheet illuminates a plane within the flow. Then, a high-speed camera captures tracer particles in two consecutive frames. The corresponding particle displacements between the two images are calculated using cross-correlation algorithms. The velocity at each point in the plane is calculated by dividing the displacement by the camera frame interval. Conventional 2D PIV illuminates a single plane with a laser sheet aligned perpendicularly to the imaging direction to detect particle displacements [12].

PIV, developed in the 1980s [13], represents a synthesis of advancements in modern materials, digital imaging, laser technology, and image analysis. As a transient, multi-point, non-contact technique, PIV enables precise measurement of transient flow fields within planar domains while simultaneously visualizing flow field structures. Without interfering with flow motion, it provides an optimal data foundation for both qualitative characterization and quantitative investigation of fluid dynamics [14]. The direct predecessor of PIV is Laser Doppler Velocimetry (LDV). Following LDV, this technique has become another laser-based digital analysis system for scientific research and industrial measurement applications.

The origins of PIV can be traced to Laser Speckle Velocimetry (LSV). Both PIV and LSV share identical technical principles and data processing methodologies when measuring flows [15]. LSV operates based on Young’s optical interference principle: when laser beams illuminate scattering particles, the particles generate correlated scattered light. Through double-exposure photography, paired speckle patterns (“speckle pairs”) are captured, analogous to double-slit interference in optical experiments. Velocity computation involves taking the ratio of slit separation distance to exposure duration [16]. Besides light-particle interactions, PIV measurements also depend on the tracing fidelity of seeding particles. Particles with density matching that of the fluid exhibit motion consistent with the flow, enabling their velocity measurements to characterize the fluid motion [17,18]. In PIV implementation, tracer particles are dispersed over the measurement area, illuminated by a light source, and their reflected light is captured by sCMOS/CCD cameras. Sequential images of the measured area are processed through image analysis systems, yielding instantaneous particle velocity corresponding to the fluid. Compared to traditional measurements, such as Pitot tubes, hot-wire/hot-film anemometers, and laser/ultrasonic Doppler velocimeters, PIV offers non-contact flow field measurement with high precision and spatial resolution. These advantages have made PIV a widely used flow measurement technology in fluid dynamics and aerodynamic research [19].

### 2.2. Particle Tracking Velocimetry (PTV)

PIV and PTV represent two main approaches in non-contact volumetric measurement techniques. These two techniques have different requirements for particle density. PIV estimates local velocities by performing cross-correlation operations on interrogation windows of sequential images, yielding average particle displacements. Notably, PIV algorithms prioritize the motion patterns of larger and brighter particles in local velocity estimation [20]. In contrast, PTV directly tracks individual particles across image sequences. At low seeding densities, PTV takes less time than PIV to obtain the velocity vector, though this advantage reverses at high densities [21].

PTV identifies and matches individual particles among consecutive frames, which determines their displacements to resolve localized flow velocities. Early PTV methods tracked particles by tracing four or more consecutive sequential images while periodically sampling, adopting trajectory length and deviation optimization schemes [22]. The distinction between PIV and PTV lies in their image post processing. PIV relies on statistical averaging within interrogation windows; PTV is specifically adapted for conditions where low-concentration tracer particles are measured. PTV enables individual particle trajectory reconstruction and derives the velocity vectors directly from discrete particle motions. PTV implementation typically requires seeding flows with sparsely distributed, larger-sized tracer particles. Compared with the spatial averaging PIV method, PTV delivers enhanced measurement accuracy and direct visualization of particle trajectories. This characteristic makes PTV particularly advantageous in scenarios of demanding high-resolution, Lagrangian-based flow field characterization. PTV uniquely resolves discontinuous flows, where PIV fails. In quantum fluids like superfluid helium [23], PTV successfully resolves discrete vortices, whereas PIV generates physically meaningless vector fields due to the absence of a continuum.

PTV presumes correspondence between tracer particle displacement and the flow field velocity at their respective positions. A pulsed laser illuminates a two-dimensional plane within the measured area, and then the reflected light of particles is obtained to determine their spatial positions. By analyzing sequential image frames, the displacement of individual particles is calculated. The velocity of each particle location is the displacement divided by the exposure time intervals. Synchronizing these particle velocities reconstructs the complete flow field motion information.

### 2.3. Fundamentals of Color-Coding

The 3D-3C PIV techniques have been practiced in engineering applications over the past two decades to address the growing demand for high-dimensional flow field characterization. Among these, stereoscopic PIV [24,25,26] and tomographic PIV [27,28,29] stand out as representative and widely adopted methods. Stereoscopic PIV employs two cameras positioned at a defined angle to capture images of the same measurement region. After performing cross-correlation calculations on the paired images to obtain the 2D velocity field, the out-of-plane velocity component (third dimension) is reconstructed using particle displacement information extracted from the parallax between the two camera views, typically via a projection reconstruction algorithm [30]. Callaud et al. [31] evaluated stereoscopic PIV’s superiority and quantitatively verified its back-projection algorithm. Their work further evaluated reconstruction accuracy based on angular displacement magnitudes. Stereoscopic PIV is commonly applied in liquid flow measurements and industrial wind tunnel studies [32]. Tomographic PIV, which is structurally similar to stereoscopic PIV, differs in that it requires reconstruction of a 3D particle field from 2D particle images using optical tomographic reconstruction algorithms. A 3D cross-correlation algorithm analyzes the reconstructed particle field and extracts three-dimensional flow motion information. Initially proposed by Elsinga et al. in 2006 [33], tomographic PIV was later optimized by Silva et al. in 2013 [34], who enhanced reconstruction accuracy through image-matching-based techniques. Compared to stereoscopic PIV, tomographic PIV offers greater implementation simplicity and higher reconstruction precision. However, it necessitates multi-camera arrangements at diverse angles to capture particle images, which introduces significant practical challenges. Specifically, spatial constraints for camera placement, complex alignment procedures, and calibration difficulties limit its applicability in flow measurements. Both techniques demonstrate unique advantages: stereoscopic PIV provides practical simplicity for specific engineering scenarios, while tomographic PIV achieves superior spatial resolution at the cost of increased experimental complexity. These advancements collectively address critical needs for 3D flow diagnostics in modern fluid dynamics research.

The need for multiple cameras induces complexity to this setup, prompting many researchers to seek cost-reduction strategies. Cierpka and Kähler [35] comprehensively survey 3D-3C velocimetry techniques in microfluidics, encompassing both multiple- and single-camera approaches. Subsequent research advances single-camera strategies such as using a three pin-hole aperture [36,37], image splitters producing multiple views on a single sensor [38,39,40,41], defocused PTV [42,43], optical aberrations [44], scanning laser sheets or volumes [45,46], light-field (LF) cameras [47,48,49,50], and color-encoded illumination. These approaches, however, reduce the effective resolution and aspect ratios in volumetric reconstructions (x, y, z directions), consequently diminishing depth and temporal resolution. To continuously improve measurement accuracy, many scholars have conducted further research and exploration on basic methods. The spiral phase mask shadow imaging proposed by Teich et al. supports large-volume [51], single-exposure 3D flow field measurement, but its low resolution makes it suitable for macro-turbulence analysis. The double-helix point spread function (DH-PSF) technology proposed by Bilsing et al. achieves higher precision through phase modulation and adaptive optics [52]. These innovations complement the microfluidic methods reviewed by Cierpka and Kähler [35].

Color-encoded PIV is a three-dimensional velocimetry technique that integrates chromatic 3D reconstruction with 3D optical flow algorithms. By encoding particle depth positions in three-dimensional flows using color information and combining this with 2D positional data from images, this method reconstructs a 3D velocity vector field using just one camera. Compared to tomographic PIV, color-encoded PIV features a simplified experimental setup, reduced spatial constraints, and enhanced adaptability to diverse real-world measurement environments. The development of color-encoded PIV dates back to 1992, when Smallwood et al. [53] pioneered a dual-color PIV system to study complex flow fields in combustion phenomena. This approach reduced computational demands for 2D velocity reconstruction by leveraging spectral differentiation [54]. In 2003, De Ponte et al. [55] advanced the methodology by proposing a tri-color PIV system for cyclic flow analysis. Their technique applied three-color pulsed illumination at two distinct time intervals, enabling the acquisition of three velocity fields from a single image through cross-correlation algorithms. Their innovative work offered both the potential for enhanced precision in regions with significantly varying velocities, such as separated flow fields, and an additional method for data validation.

**Figure 1:**
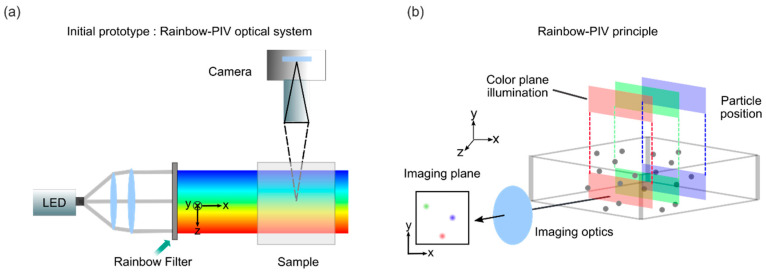
(**a**) Initial prototype: Rainbow-PIV optical system. (**b**) Rainbow-PIV principle. Reprinted with permission from reference [59].

Rainbow PIV, an advanced implementation of color-encoded PIV, facilitates three-dimensional flow characterization through spectral–spatial multiplexing. As illustrated in Figure 1a, the optical system comprises two major components: (1) A rainbow illumination module generates spectrally graded light planes via a linear variable wavelength filter to produce clean spectral planes across the sample volume. (2) A monocular imaging system oriented perpendicular to the illumination axis captures scattered light from particles within the chromatic measurement volume. Classical PIV consists of imaging tracer particles seeded into fluid to analyze the position and velocity of each tracer particle [56]. Figure 1b demonstrates the core principle of chromatic position encoding. Lateral coordinates (x, y) are resolved through conventional 2D spatial imaging. Depth (z) is encoded via wavelength-dependent illumination, where particle positions along the optical axis correlate with detected spectral signatures. This configuration gains several advantages to single-camera 3D velocimetry: simultaneous acquisition of spatial and spectral data enables dynamic 3D flow visualization to realize real-time 3D reconstruction; dense 3D particle trajectories are resolved through time-series analysis of spectral–spatial correlations for high temporal resolution; and the hardware is simplified by eliminating multi-camera synchronization and complex calibration procedures inherent in stereoscopic/tomographic PIV. Demonstrated applications include turbulent flow characterization and vortex dynamics analysis, where Rainbow-PIV successfully resolves complex 3D velocity fields with micron-scale spatial resolution [57,58]. The technique’s compatibility with conventional PIV hardware and enhanced adaptability to confined measurement environment positions make it a promising tool for advanced fluid diagnostics in both research and industrial settings.

## 3. LED-Based Illumination Systems

### 3.1. Technical Principles

In Rainbow PIV, the most common modality employs an optical setup using LEDs to generate rainbow light to encode particle depth into a large depth-of-field color image. These images are then jointly optimized to determine particle positions and velocity fields using a new reconstruction algorithm. The Rainbow PIV is based on LED illumination, which is provided by a collimated and filtered white light source, establishing a linear wavelength–depth relationship throughout the flow domain. Here, particles function as narrowband point sources with axially varying emission wavelengths. The optical configuration additionally integrates a diffractive optical element (DOE). It is a Fresnel phase plate created via micrometer-scale surface patterning on a glass substrate. This component implements wavelength-selective focusing for the camera, then enables fully focused imaging of particles in the measured area. Reconstruction entails spatial localization via spectral decoding of particle images and spatiotemporal tracking for 3D velocity field derivation, thereby establishing comprehensive 3D-3C velocity vectors.

### 3.2. Application Cases

**Figure 2:**
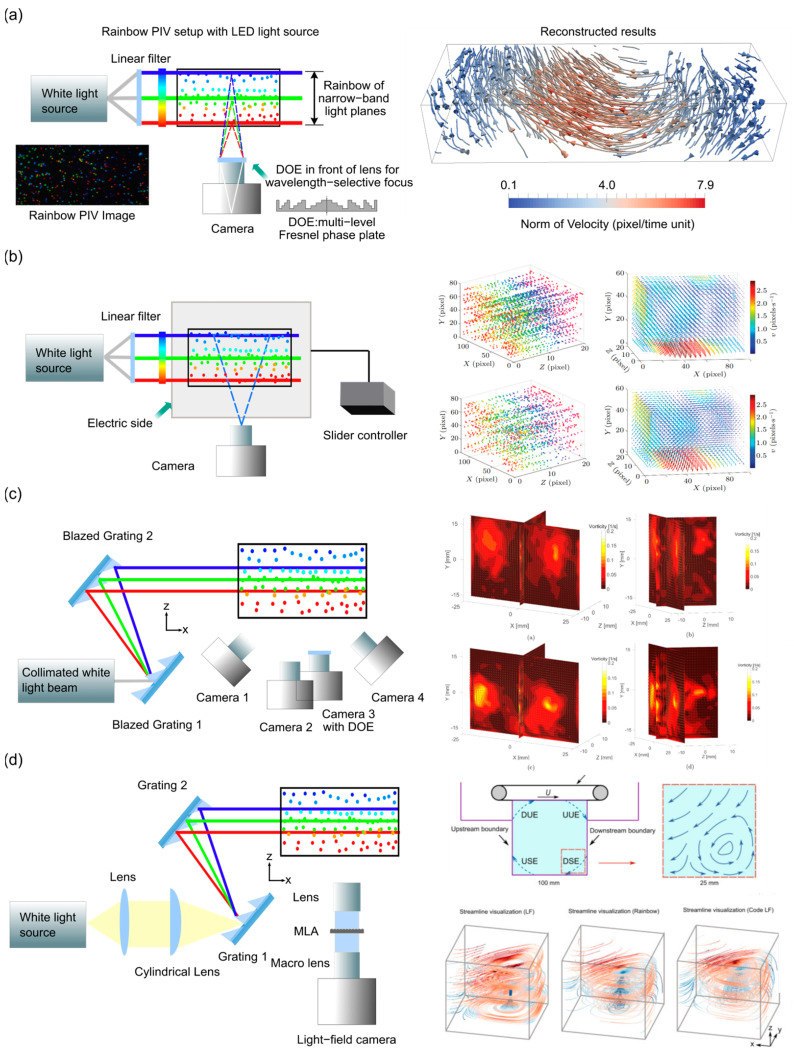
Systems and applications of LED-based illumination. (**a**) The measurement setup for Rainbow PIV is illustrated (**left**). Velocity vectors are discretized across a structured lattice covering the flow domain, with visualization of the tracers of particles employed for spatial representation (**right**). Reprinted with permission from reference [60]. (**b**) The principle of the 3D color PIV experiment is depicted (**left**). Visualization of 3D particle distribution and estimated velocity field of double-vortex experiment is shown (**right**). Reprinted with permission from reference [63]. (**c**) Schematic diagram for the experimental setup using two blazed gratings to generate a size-controlled rainbow (**left**). Reconstructed flow vectors and vorticity magnitude are presented (**right**). Reprinted with permission from reference [65]. (**d**) Schematic drawing shows the excitation beam (**left**). Schematic drawings of setups for square lid-driven cavity flow measurement and the streamline visualization obtained from light-field, Rainbow, and Code light-field PIV are presented (**right**). Reprinted with permission from reference [66].

Xiong et al. [60] proposed a novel Rainbow PIV method for dense 3D velocity mapping in microfluidic flows. The main challenge addressed in this study is the limitation of conventional PIV techniques in acquiring volumetric velocity information with high spatial resolution, especially in dense particle environments. The researchers developed an experimental system based on color-encoded illumination and single-camera imaging, which enables single-frame retrieval of particles in 3D space by exploiting the wavelength-dependent penetration depth of colored light in a dye-filled medium. The reconstruction algorithm employs an image formation model to obtain particle 3D coordinates per frame. Velocity fields are subsequently derived from spatiotemporal frame sequences via an optical flow approach [61,62]. The experimental setup includes a broadband white light source, a prism-based wavelength dispersion system to create a spatially distributed rainbow illumination, a microfluidic channel filled with a dye solution to modulate light absorption, fluorescent tracer particles, and a camera (Figure 2a, left). The key to this system is using polychromatic light to encode depth information into the particle color, since particles at different depths reflect corresponding wavelengths due to differential absorption and appear in different colors in the captured image. A pre-calibrated map linking color to depth is used to recover the 3D position of each particle. The authors validated their methods using both simulated and experimental flows, demonstrating accurate 3D reconstruction of velocity fields in microchannels. Through PIV technology analysis and calculation, velocity vector data that can reflect the instantaneous state of the flow field are generated at regularly distributed grid points. These discrete velocity vectors, like coordinate points in the flow field, provide a fundamental basis for subsequent streamline plotting. The finally presented streamlines are obtained through spatial integration operations on these velocity vectors. The reconstructed flow patterns, such as the parabolic velocity profile in a straight channel and the complex secondary flow structures in curved geometries (Figure 2a, right), align well with theoretical predictions. These results indicate that Rainbow PIV offers a promising single-camera technique for dense volumetric velocimetry in microflows, overcoming many limitations of conventional 3D PIV approaches in terms of optical access, complexity, and spatial resolution.

Shan et al. [63] devised an enhanced Rainbow PIV method integrating fast Fourier transform (FFT) cross-correlation with the Horn–Schunck (HS) optical flow method, achieving accelerated velocity reconstruction without compromising displacement. The research specifically focuses on the limitations of the Rainbow PIV algorithm [64], particularly its high computational cost, by proposing a hybrid method that reduces reconstruction time by over 20% without compromising accuracy. The experimental system (Figure 2b, left) employs a single-camera setup with color-encoded illumination for particle depth information. An LED-generated white light beam passes through a linear spectral filter to create a volumetric colored illumination and illuminate the tracer particles suspended in a sample chamber, where their depth positions are encoded via the color of reflected light. A Fourier lens simultaneously capturing both spatial and color-depth information recorded these color particle images. The efficient and cost-effective system avoids the complexity of multi-camera arrangements.

To validate the method, both simulated and experimental flow fields were examined. For simulations, the researchers used a Rankine vortex model with varying particle velocities and densities, allowing quantitative evaluation under controlled conditions. The reconstructed 3D velocity fields closely matched ground-truth data with average errors nearly identical to those from the conventional method. Notably, the proposed algorithm consistently achieved a 20–25% reduction in computation time. For experimental validation, single- and double-vortex datasets were processed (Figure 2b, right). The reconstructed flow fields from the proposed hybrid method displayed structures and patterns that were nearly identical to those from Rainbow PIV, as confirmed by high structural similarity index (SSIM) values of 0.9815 (single vortex) and 0.9872 (double vortex). Visualization of the reconstructed 3D flow fields showed clear vortex structures with smooth gradients, with the maximum velocity magnitude reaching over 3.5 pixels/s in single-vortex experiments. The hybrid algorithm completed reconstruction in approximately 280 s, outperforming Rainbow PIV.

To address axial resolution constraints in single-camera Rainbow PIV, Xiong et al. [65] proposed a depth-super-resolved Rainbow PIV method. Their study focused on enhancing depth accuracy while preserving system simplicity, with direct comparison against a benchmark four-camera Tomo-PIV setup under identical experimental conditions. The experimental setup combined both Rainbow PIV and tomographic PIV configurations (Figure 2c, left). Four high-resolution color cameras were positioned around an octagonal tank for tomographic PIV measurements, while a central bottom camera equipped with a customized DOE was used for Rainbow PIV. The illumination system generated a spatially collimated rainbow beam by two blazed gratings and an LED-generated white light source, creating a 25 mm thick chromatic light sheet that encoded depth through wavelength variations. The system used white polyethylene microspheres as tracer particles and a vortex ring generator to produce complex flow structures. The reconstruction framework employed a joint optimization scheme that simultaneously estimates 3D particle distribution fields and 3D-3C velocity fields across sequential frames. Depth super-resolution was achieved by interpolating the camera’s point spread functions (PSFs) across 100 discrete axial levels, allowing sub-voxel particle localization. This method enhanced axial accuracy without additional hardware. Flow reconstruction results showed that Rainbow PIV could recover vortex ring structures with accuracy comparable to tomographic PIV in both qualitative and quantitative terms (Figure 2c, right). Notably, Rainbow PIV achieved lower divergence in the reconstructed flow fields, satisfying incompressibility constraints more effectively than tomographic PIV. The improved axial resolution reduced errors in the z-component of velocity by more than 50% compared to those of the original Rainbow PIV. Additionally, Rainbow PIV demonstrated robust performance under low seeding density conditions (as low as 0.005 particles per pixel), outperforming tomographic PIV in scenarios with poor optical access or sparse tracer distributions. This work established depth-super-resolved Rainbow PIV as a compact and physically consistent alternative to traditional multi-camera volumetric systems, especially in constrained experimental configurations.

Xing et al. [66] proposed a novel high-resolution 3D PIV method termed Code light-field PIV, combining color-depth encoded illumination with light-field imaging. The study addressed the critical limitation of poor axial resolution in conventional light-field PIV by introducing a single light-field camera to capture a structured rainbow illumination beam encoded along the depth axis. This compact and cost-effective system enabled high-fidelity 3D-3C flow measurement. The experimental system comprised a broadband white LED source (445–695 nm) diffracted by a pair of blazed gratings to generate a rainbow beam with spatially encoded color along the axial direction (Figure 2d, left). The beam illuminated a 25 mm deep volume, matching the imaging region. The detection system featured a light-field camera with a microlens array and a 4.2 MP CMOS sensor. The camera recorded color light-field images containing both spatial-angular and spectral-depth information. A two-step reconstruction method was employed: firstly, standard light-field refocusing generated a coarse 3D particle field; secondly, an iterative reconstruction algorithm used the known color-depth calibration map to refine the axial positions of particles, significantly enhancing depth resolution. The process achieved axial resolution of 200 μm across 125 depth layers within 25 mm. To evaluate system performance, the authors imaged a classical square lid-driven cavity flow (100 × 100 × 100 mm^3^) at Reynolds number 3286, focusing on the downstream secondary vortex (DSE) region (Figure 2d, right). The reconstructed volume (25 × 25 × 25 mm^3^) was divided into 250 × 250 × 125 voxels, achieving 100 μm lateral and 200 μm axial spacing. The velocity field was derived using an optical flow model optimized with divergence-free and correspondence terms. The results clearly captured the main vortex, secondary vortex, and separation regions. Particle trajectories were analyzed by frame-to-frame matching, confirming the reconstructed flow structures. Code light-field PIV outperformed conventional light-field PIV and Rainbow PIV by both significantly improving accuracy and reducing divergence errors (<0.008 s^−1^), indicating high physical consistency in incompressible flows.

### 3.3. Merit–Demerit Analysis for LED-Based Illumination Systems

LED-based systems have emerged as versatile solutions for particle velocimetry due to their adaptability and low operational costs. These systems have significant advantages in energy efficiency and portability. LEDs typically consume less than 10 W, enabling integration into portable devices such as handheld microfluidic analyzers. Unlike lasers, LEDs generate minimal heat (<0.5 °C temperature rise during continuous operation), making them suitable for temperature-sensitive biological applications, such as live-cell imaging in microchannels. But LED-based systems have limitations such as spatial resolution constraints. The low spatial coherence of LEDs limits their resolution to 1–5 μm, restricting applications in nanoscale flow analysis. In terms of temporal performance and environmental sensitivity, although pulsed LEDs achieve frequencies up to 10 kHz, they remain inferior to lasers for capturing ultrafast phenomena like shockwave propagation. In outdoor or high-ambient-light environments, LED signals suffer from signal-to-noise-ratio (SNR) degradation, necessitating spectral filtering or synchronized shuttering to decrease the noise.

## 4. Projector-Based Illumination Approaches

### 4.1. Working Mechanism

Aguirre-Pablo et al. [67] devised intensity PTV with structured monochromatic illumination. The method used a liquid crystal display (LCD) projector as the light source. A rainbow gradient was projected by the LCD projector, altering the hue along the camera’s depth axis. Because the LCD projector emitted divergent light, which was inherently devised to illuminate large spaces, a collimation lens was applied to the system to generate a parallel color beam to fix the color-depth correspondence. The particles seeded into a test fluid were illuminated by a color beam and captured by the camera. The depth of the particle scattered light followed a color-dependent manner under color gradient illumination. Consequently, the in-plane (x and y) and out-of-plane (z) coordinates of the particles were, respectively, ascertainable from the image pixel coordinates (i and j) and the detected color. Color PTV, characterized by its straightforward yet efficient principle, enabled a broad measurable depth and accommodated high particle image density, all without the necessity for intricate setups.

### 4.2. Typical Implementation Examples of Projector-Based Illumination Approaches

**Figure 3:**
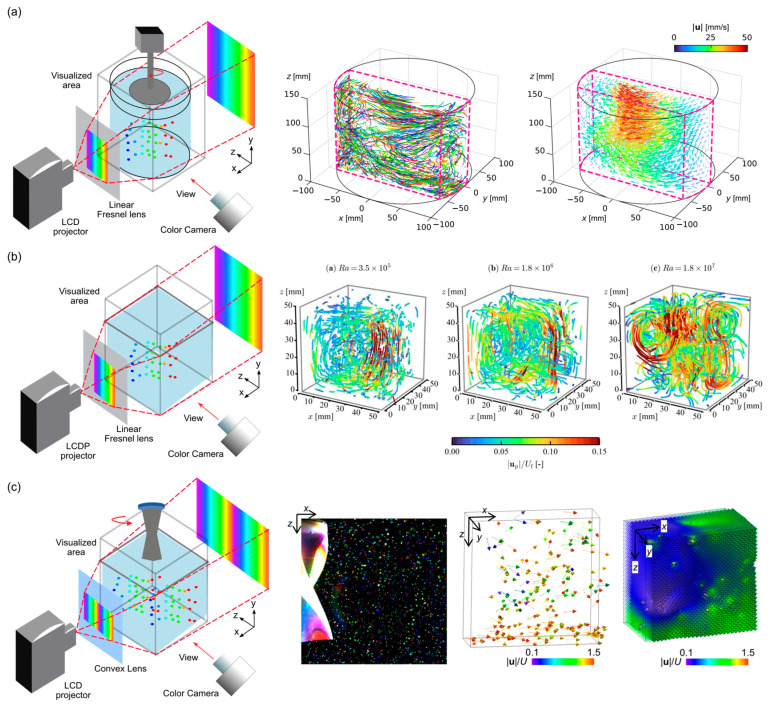
Systems and applications of projector-based illumination. (**a**) Schematic of the experimental setup for the rotating flow measurements (**left**) and the reconstructed 3D flow field in a rotating flow induced by an impeller (**right**). Reprinted with permission from reference [68]. (**b**) Schematic illustrations of the experimental setup (**left**) and 3D particle trajectories obtained for Ra = 3.5 × 10^5^, Ra = 1.8 × 10^6^, and Ra = 1.8 × 10^7^ (**right**). Reprinted with permission from reference [69]. (**c**) Two-cycle rainbow color PTV with defocusing technique. Schematic diagram of facility setup (**left**) and the processes to obtain 3D–3C instantaneous velocity field (**right**). Reprinted with permission from reference [70].

Numerous hurdles need to be overcome to guarantee sufficient precision during the practical implementation of the projector-based illumination strategy. Noto et al. [68] addressed the in situ calibration of the correlation from color to depth, which was a key technical challenge in the development of color PTV for accurate three-dimensional flow field reconstruction. Conventional methods relied on pre-calibrated depth-color maps, which were highly sensitive to changes in imaging geometry, lighting conditions, and optical aberrations. These challenges made them impractical for dynamic experimental setups. To overcome this, the authors proposed an in situ calibration technique that reconstructed the color-to-depth mapping directly during experiments, eliminating the need for a precisely controlled calibration environment. As shown in Figure 3a (left), the experimental setup included several major components: a rectangular vessel with a cylindrical annulus seeded with tracer particles; a propeller-driven impeller generating a rotating flow field as the validation target; a consumer-grade LCD projector producing a rainbow-colored beam, collimated by a Fresnel lens illuminating a 60 mm-thick volume; and a single CMOS camera capturing side view particle images. The full-field volume measured was approximately 200 × 60 × 150 mm^3^. Depth encoding was achieved through the color gradient, and particle depth positions were inferred from their RGB values.

To calibrate color-to-depth mapping, the authors used a series of sheet-color illumination patterns—thin rainbow slices projected through the same setup—to illuminate particles at known depths. Over 40 depth layers, more than 200,000 particle color samples were recorded. A neural network was trained using both particle position (i, j) and color matrices (3 × 3 RGB values) to predict the depth (z) coordinate. Three-dimensional particle tracking was performed by linking 2D image-plane trajectories and correcting the depth information using temporal smoothing and robust regression. Validation in a complex rotational flow showed that the reconstructed 3D flow field captures key structures like toroidal vortices (Figure 3a, right). Comparison with conventional PIV shows good agreement in both vertical and horizontal cross-sections. Although some edge-region discrepancies still existed due to sparse particle distribution, the overall structure and velocity magnitudes were well reproduced. This study demonstrated that the in situ calibrated color PTV method offered an accurate and low-cost alternative for 3D-3C flow measurements, especially valuable for experimental setups with limited optical access and high complexity.

Subsequently, Noto et al. (2023) [69] proposed a cost-effective and practical 3D color PTV method enabling 3D-3C flow measurements with a single color camera and a consumer-grade LCD projector. This method offered a simpler and more accessible solution for fluid dynamics research. A key improvement was the in situ “digital slit calibration”, which constructed the color-to-depth mapping directly within the actual experimental environment, and thereby mitigated the effects of optical distortions and environmental variability. The experimental setup centered around a Rayleigh–Bénard convection system with a 50 mm^3^ cubic water cell, heated from below and cooled from above. Tracer particles were illuminated by a collimated rainbow beam generated by the LCD and a Fresnel lens, creating a depth-encoded color field (Figure 3b, left). The particle images were documented with a color camera that was arranged orthogonally to the color gradient. The RGB brightness values of the particles were linked to their depth positions via a calibration process involving digitally projected slit rainbow patterns through the flow domain. An artificial neural network (ANN) was trained using these data to perform color-to-depth conversion, accounting for in-plane coordinates and local color matrices of particles. The authors applied this technique to measure flow structures at various Rayleigh numbers (Ra = 3.5 × 10^5^ to 1.8 × 10^7^), spanning laminar to soft turbulent states. Their method successfully reconstructed 3D particle trajectories and velocity fields, revealing isotropic features in the turbulent regime (Figure 3b, right). Statistical comparison between in-plane (x) and out-of-plane (y) velocity components demonstrated that the ANN-predicted depth positions—once temporally filtered—yield velocity statistics comparable to traditional stereoscopic techniques. Overall, this work showcased a viable, low-cost alternative to tomographic PTV, enabling efficient and accurate 3D flow visualization without the complexity of multi-camera setups. By significantly reducing hardware requirements and calibration efforts, the method made volumetric velocimetry more accessible for broader experimental applications.

Park et al. [70] developed an improved 3D color PTV method by integrating multi-cycle rainbow illumination with a defocusing imaging technique. This method addressed two critical challenges in single-camera 3D-3C PTV: the poor depth resolution caused by false color artifacts and the limited measurable depth. False colors originated from color interpolation in Bayer sensors, particularly at the edges of in-focus particle images. The experimental system included a color high-speed camera (FASTCAM Mini AX50, Photron, San Diego, CA, USA) and an LCD projector (Epson EB-W420, Suwa, Nagono, Japan) generating volumetric rainbow illumination. A convex lens collimated the illumination along the depth (z) direction. Tracer particles suspended in a viscoelastic fluid were illuminated in a tank, with particle images recorded at 750 fps.

As shown in Figure 3c (left), the system used a projector to generate a double cycle of rainbow illumination. This system encoded the measurement volume with a periodic color gradient that repeated twice along the depth (z) direction, enhancing the depth resolution and expanding the usable measurement volume without increasing system complexity. The method assigned particle depth based on both hue (color) and image size. In conventional single-cycle color PTV, each hue corresponded to a unique depth. However, the hue-depth mapping was limited by dynamic range and might suffer from poor gradient in some regions. In contrast, the two-cycle method overcomes these limitations by introducing a repeating color sequence across the depth range. Particles located at two different depths could have the same hue but differ in image size due to defocusing. By intentionally defocusing the camera lens, particle images exhibited size variation that correlated with their axial position. Thus, the ambiguity in hue could be resolved by correlating the induced size variations. For example, two blue particles in the image could be distinguished if one appeared larger, indicating its farther position in the second rainbow cycle. The synchronization of hue and defocused size enabled unambiguous depth estimation with finer resolution.

A pivotal innovation was the implementation of pattern-adaptive, moon-shaped correlation masks to effectively detect distorted particle images and their true centers under defocused conditions. The proposed technique was applied to visualize the wake flow behind a twisted Savonius turbine in a towing tank experiment (Figure 3c, right). The 3D flow field was reconstructed from four-frame particle tracking, capturing ~120 instantaneous velocity vectors per frame with sub-pixel location accuracy (~0.013U). The reconstructed flow field revealed key vortical structures: a vertical ascending flow from the bottom of the turbine and a streamwise vortex shedding downstream. These structures achieved more uniform velocity profiles and reduced turbulence intensity compared to those of a straight-blade turbine. This study demonstrated that combining defocusing imaging with multi-cycle rainbow illumination significantly enhances depth resolution and particle detectability in single-camera 3D PTV. The successful reconstruction of 3D-3C flow around a complex geometry demonstrated the method’s potential for volumetric flow diagnostics, especially in constrained or opaque optical environments.

### 4.3. Merit–Demerit Analysis for Projector-Based Illumination Approaches

Projector-based rainbow illumination has emerged as a practical and cost-effective approach for encoding depth information in 3D color PTV systems. By using commercial LCD projectors to generate continuous, spatially graded color fields, it enables single-camera volumetric flow measurements without requiring complex multi-camera setups or mechanical scanning. As demonstrated in studies above, this illumination strategy is particularly suited for experimental configurations with limited optical access and moderate spatial scales.

One of the key advantages of projector-based systems is their flexibility: illumination patterns could be digitally programmed to achieve single- or multi-cycle color gradients, sheet-by-sheet calibration, or dynamic adjustments. This allows researchers to extend the measurable depth range and enhance axial resolution. However, several limitations must be considered. The relatively low brightness of consumer-grade projectors could hinder particle visibility, especially in high-speed imaging scenarios. Additionally, color distortion due to the sensor’s Bayer interpolation often leads to false hues near particle edges, requiring advanced calibration techniques such as neural networks or defocusing compensation. The nonlinearity and non-uniformity of color distribution across the illuminated volume also necessitated careful in situ calibration. Furthermore, particle overlap and color ambiguity might become problematic under high seeding densities or poor focus conditions.

## 5. Laser-Based Illumination Architectures

### 5.1. Fundamental Theories

Conventional lighting systems for PIV also used pulsed lasers as a light source. Due to the fact that the pulsed laser discharged pulsed laser light, boasting the merits of high energy, low divergence, and good monochromaticity, the output of the conventional pulsed laser was unstable [71], which could easily affect the particle imaging. Hellwarth and McClung [72] proposed in 1961 to adjust the quality factor Q of the laser resonance cavity so that the laser could maintain a narrow width while outputting high-energy laser. Regunath et al. [73] made a breakthrough in cost-effective volumetric PIV by demonstrating dual-plane measurements using a single Nd:YAG laser. By routing 532 nm laser pulses through a fluorescent dye converter, they generated a secondary 572 nm sheet with controllable spacing down to 1 mm. With the wider application of PIV technology in fluid dynamics, aerodynamics, and other research fields, the development of lasers with both high beam quality and high stability had become an important direction of PIV research [74].

As aforementioned, either application of high-power white LED as an excitation source [75] or the LCD projector as a light source could provide low-cost and simple illumination beams. However, due to the power limitations of the LED light source, their applications were limited to low-speed flow imaging. In addition, to ensure that all illuminated particles are focused, both modalities required either customized diffractive optics to maintain a large NA or a reduced NA of the imaging lens, leading to a compromise between system budget and light harvesting efficiency. In this context, laser synthetic coding-based systems offered a completely new direction in the development of color-encoded PIV to address these limitations.

### 5.2. The Typical Implementation Examples for Laser-Based Illumination Architectures

**Figure 4:**
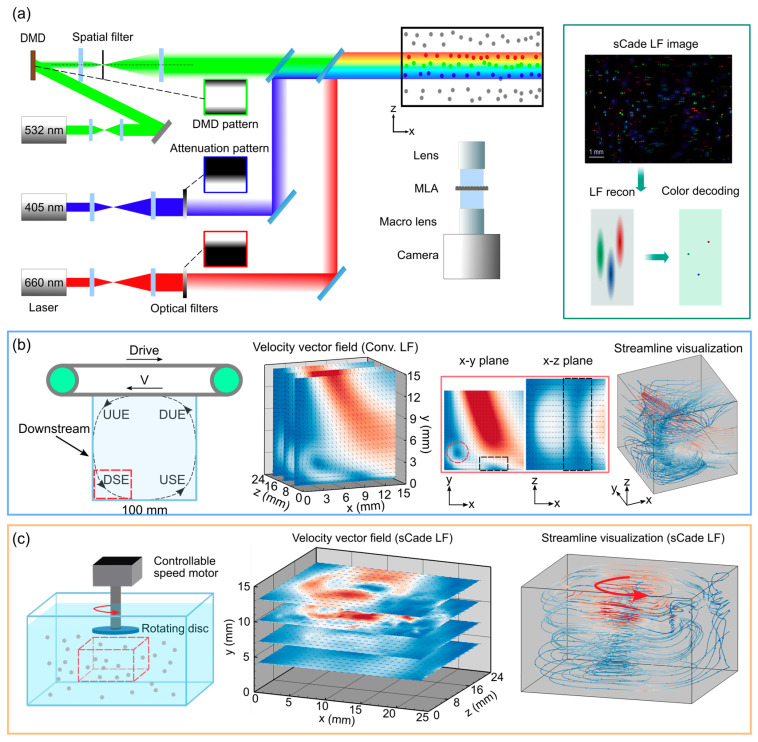
Systems and applications of laser-based illumination. (**a**) Working principle of sCade light-field PIV, schematic diagram showing the design of the imaging system. sCade LF-PIV uses RGB color lasers (404 nm, 532 nm, and 660 nm) to generate a composite beam. The beam is first intensity-encoded in the depth direction with specific patterns, then mixed and superimposed. The right diagram illustrates the light field image reconstruction process. (**b**) Measurements of sCade light-field PIV for the square lid-driven cavity flow. From left to right: the schematic diagram of the square lid-driven cavity flow measurement device, and the flow vector field obtained by sCade LF-PIV. Red circles denote the centers of the captured secondary vortices, and black boxes indicate flow separation regions. A cropped region is shown in the image for clear visualization. (**c**) Measurements of sCade light-field PIV for the vortex. From left to right: the schematic diagram of the vortex measurement device, the flow vector field obtained by sCade LF-PIV, and the streamline visualization. Reprinted with permission from reference [76].

Wang et al. [76] introduced a synthetic color-and-depth encoded (sCade) illumination technique to overcome the axial resolution limitations of conventional light-field PIV. Conventional light-field PIV systems offered snapshot 3D imaging with a single camera but suffered from elongated point spread functions (PSFs) along the depth, reducing accuracy in volumetric flow measurements. To address this, the authors developed a high-resolution sCade light-field PIV system employing laser-driven rainbow illumination and a color light-field camera. The experimental setup comprised three laser sources (405 nm, 532 nm, and 660 nm), with intensities modulated via a digital micromirror device (DMD) and neutral density filters to synthesize a depth-encoded multicolor beam. These beams were optically combined, collimated, and directed to illuminate the measurement volume. A custom-built light-field camera, equipped with a microlens array and a 4.2 MP color CMOS camera (Shenzhen MindVision Technology Co., LTD, Shenzhen, Guangdong, China), captured volumetric color images of tracer particles. Depth information was decoded from the particles’ RGB intensity ratios using a calibrated color-depth mapping, which was then refined via an ADMM-based iterative algorithm to reconstruct high-accuracy 3D particle distributions (Figure 4a).

To validate the system, the authors imaged two flow configurations: a square lid-driven cavity flow and a disk-induced vortex. For the cavity flow (25 × 15 × 24 mm^3^), sCade LF-PIV resolved both primary and secondary vortices, with reconstructed velocity fields aligning closely with CFD simulations (Figure 4b). In contrast, conventional light-field PIV failed to resolve secondary structures. In the disk vortex case (Figure 4c), where axial resolution was critical, sCade light-field PIV captured complete 3D vortex structures with minimal divergence error (<0.0008 s^−1^) compared to conventional methods (>0.0025 s^−1^). Quantitatively, the system improved axial localization resolution by 29 times and significantly reduced measurement error across all flow components. This improvement enabled accurate 3D-3C flow measurements in complex, fast, or constrained environments. Moreover, the application of lasers has enabled high-intensity illumination for high-speed imaging, extending utility to turbulent or unsteady flows. This work demonstrated that sCade light-field PIV offered a powerful, high-resolution alternative to conventional light-field PIV and Cade PIV methods, particularly by combining the spatial capture of light-field imaging with the depth discrimination of chromatic encoding.

### 5.3. Feature Analysis

Laser-synthesized rainbow illumination provides a high-performance approach for encoding depth information in color-encoded PIV and PTV systems. Compared to projector- or LED-based illumination, laser systems provide significantly higher brightness and superior directional coherence, making them ideal for high-speed imaging and time-resolved volumetric flow measurements. By combining multiple discrete-wavelength lasers (405 nm, 532 nm, 660 nm) and modulating their spatial intensities using a digital micromirror device (DMD), researchers construct programmable, spectrally controlled illumination patterns that encode depth through RGB color ratios. This approach offers several advantages. First, the high luminous intensity of lasers enables clear particle visualization even at high frame rates, which is crucial for studying turbulent or transient flows. Second, the narrowband spectral characteristics of lasers ensure stable and accurate color-to-depth mapping. Third, the use of the DMD allows for spatially tailored intensity profiles, enhancing the uniformity and resolution of depth encoding. As aforementioned, this method achieves axial localization resolution up to 29 times higher than conventional light-field PIV methods. Furthermore, the strong contrast and low background noise of laser illumination the improve signal-to-noise ratio, which is particularly beneficial in optically constrained or noisy environments.

However, laser-based systems also demonstrated several limitations. The optical setup is complex, requiring precise alignment of multiple laser beams, filters, mirrors, and collimating optics. System calibration and maintenance could be time-consuming. Additionally, the discrete nature of laser wavelengths limits the smoothness of the color gradient, potentially reducing depth encoding fidelity. Safety concerns related to laser exposure must also be addressed, along with potential speckle noise that could degrade particle image quality. In summary, laser-based rainbow illumination provides an advanced, high-resolution tool for color-encoded PIV applications, especially in high-speed or high-precision environments. Yet, its complexity and cost may limit its use in standard laboratories without specialized equipment or expertise.

## 6. Comparative Analysis and Future Trends

### 6.1. Performance Comparison for Variant Illumiantion Strategies

Continual improvement in hardware and image processing methods has significantly enhanced the accuracy, dynamic range, and computational speed of color-encoded PIV. While methodological innovations drive the development of color-encoded PIV technology, practical application still requires overcoming application-centric barriers, such as hardware portability, operational robustness, and modular design, to ensure the utility of measurements in real-world engineering applications. This technique is now widely used in fields such as fluid mechanics, aerodynamics, and life sciences, with expanding applications as a critical method for modern fluid measurement.

The performance of light-based particle velocimetry systems varies significantly with the illumination strategy. LED-based systems offer notable advantages in energy efficiency and cost-effectiveness with typical power consumption below 10 W, making them suitable for portable setups. However, their limited spatial resolution and pulse frequency restrict applications in high-speed or high-precision measurements. Their low luminous flux also necessitates shielding in bright environments. Projector-based illumination excels in large field-of-view applications, achieving uniform illumination across areas exceeding 100 × 100 mm^2^. Apart from that, projectors suffer from dynamic response lag due to refresh rate limitations, optical efficiency losses of 30–50% from diffractive splitting, and frequent calibration requirements to correct geometric distortions. Laser-based systems, on the other hand, provide unparalleled spatiotemporal resolution (sub-micron and nanosecond-scale control) and superior SNR due to their monochromatic coherence, making them indispensable for turbulent flow analysis. Yet, their complexity in optical alignment and safety risks from high-power operation pose significant challenges. Thus, the choice of illumination depends on a trade-off between resolution, speed, cost, and operational constraints, while emerging hybrid systems seek to combine their strengths. We summarize three different illumination strategies in Table 1.

### 6.2. Emerging Directions

Future progress in rainbow-light particle velocimetry would be driven primarily by innovations in laser-based or hybrid illumination systems that synergize the strengths of LEDs, projectors, and lasers. Next-generation ultrafast pulsed lasers with femtosecond-scale resolution could enable unprecedented temporal analysis of turbulent flows, while wavelength-tunable fiber lasers might allow dynamic adaptation to diverse particle tracers. The integration of multi-beam laser arrays promises to expand measurement volumes without sacrificing spatial resolution, addressing a key limitation in volumetric velocimetry. Their flexible modulation capabilities, such as programmable RGB wavelength switching, enable dynamic color-coding for flow tracking, with the current focus on three primary color channels (red, green, blue) representing a deliberate trade-off for cost-effectiveness in color-depth encoding PIV systems rather than a technical constraint—these channels sufficiently meet basic encoding needs for low-cost applications, though systems with more than three channels (e.g., covering 450 nm, 550 nm, 650 nm, 800 nm) could be constructed using ultraviolet/infrared-sensitive cameras and broader-range filters/dichroic mirrors, albeit at significantly higher cost. Additionally, combining LED arrays with laser speckle patterns could enable high-speed, large-FOV measurements while mitigating respective limitations (e.g., cost and complexity). The frame rate of the camera restricts the imaging speed. Employing an ultrafast camera is a viable approach to enhancing the imaging frame rate for capturing transient flow images [77,78,79,80]. Miniaturization and embedded systems might extend the applications from lab-scale studies to industrial monitoring. Standardized calibration protocols and open-source algorithms would be critical to generalize these technologies. For color decoding, several algorithmic challenges remain to be addressed. Spectral overlap between emitted and reflected light can cause signal crosstalk, reducing the distinguishability of depth-related color features. To tackle these issues, deep learning-based decoding techniques have shown promising potential [81,82,83]: end-to-end reconstruction networks can learn complex mapping relationships between raw color signals and depth information, effectively mitigating the impact of spectral overlap; attention mechanisms embedded in these networks can focus on noise-robust features, enhancing the stability of decoding under noisy conditions. When it comes to the speed of data processing, implementing methods grounded in deep learning could also quicken the reconstruction of the displacement field [84], making high-resolution volumetric imaging attainable. The application of existing color-encoded illumination technology in dynamic hydraulic systems is still limited by decoding efficiency and noise interference, while the advantages of deep learning algorithms in image feature extraction have been verified in static scenarios. In the future, focus can be placed on exploring lightweight neural network architectures and multimodal data fusion strategies to meet the monitoring requirements of more dynamic flow fields.

## Figures and Tables

**Table 1:** A brief comparison of various illumination strategies.

Parameters	Typical Resolution	System Cost	Environmental Adaptability	Control Complexity	Typical Application
LED	1–5 μm	Low	Low	Low (dimming)	Stirring disturbance, vortex ring, square cavity driving, etc.
Projector	10–50 μm	Medium	Medium	Medium (programming)	Cylindrical rotating flow, flow driven by rotating impellers, wake flow of twisted turbine shedding, etc.
Laser	0.1–1 μm	High	High	High (optical path calibration)	Vortex driven by motor rotors, turbulence research, etc.
